# Extracts and Gleanings

**Published:** 1875-03

**Authors:** 


### Extracts and Gleanings.

*The Dispersion of Tumors by Puncture.*—Dr. Cameron observes that those familiar with the East are aware that, from time immemorial, the native hakins have been accustomed to attempt to bring about the absorption of the enlargements of liver and spleen, so common in hot malarious countries, by the use of puncture with long sharp stillets of considerable thickness. Twining, in his work "On the Diseases of Bengal," mentions the practice. Dr. Cameron states that he has never followed it for the purpose of procuring dispersions of such enlargements, but that he has frequently seen those of the liver disappear rapidly after repeated plunges of an ordinary hydrocele trocar when seeking unsuccessfully for suspected abscess, and he never found in any instance inflammatory or any other bad symptoms produced by such operations, strange as it may appear to those unaccustomed to perform them. What he wishes particularly to draw attention to is, that other enlargements besides those of the liver and spleen may be made to disappear by puncture. Nothing is more tedious than those chronic glandular swellings which in strumous subjects, often in hot countries follow upon trifling causes, such as angry mosquito bites, riding a rough bucking horse, over-exertion, or a strain in cricketing, and so forth. He states that he has known an officer laid up for many months and ultimately invalided, with a large mass of indurated enlarged glands occupying the whole inguinal region, and resisting all the recognized routine of treatment. Accident showed him that deep puncture of such masses with a common lancet held at right angles to the swelling, and pushed down to its bottom, will often cause absorption to set in and proceed rapidly. The first case in which this occurred to him was one of n mercantile gentleman, disabled by a mass of swollen inguinal glands almost as hard as a board, and resisting all treatment. This patient's loss of time at office was a very serious matter to him, and, influenced by his despairing impatience, Dr. Cameron plunged a lancet perpendicularly into the mass as far as it would reach. The point came out tinged with matter, and hard pressure brought up a little cheesy, ill-formed pus, but no discharge whatever followed, and absorpti n set in and proceeded rapidly. This and several other cases which he mentions have led him to think that this mode of puncture might be found to bring about the dispersion of such growths as fibrous tumors of the uterus; and reasoning from the non-supervention of evil symptoms after repeated and deep puncture of the liver with a trocar, he sees no ground for fearing to puncture with a small stillet such a fibrous uterine tumor as is often plainly felt through the abdominal parietes, and he thinks puncture through them less likely to be followed by evil consequences than puncture per vaginum, owing to the exclusion of air.—*Lancet.*

*Case of Chronic Arsenic Poisoning.*—Holm, in the *Upsala läkareföören förhand,* describes a dozen cases of arsenic poisoning from the wall-paper, lamp-screens and curtains of dwelling-houses. In these cases the etiology was evident, and the symptoms very distinct and characteristic. The latter were chiefly the following: Headache, with a sensation as of a ligature tightly embracing the head; giddiness and fainting; occasionally a faltering gait and a fog before the eyes; the latter were often red and painful; nausea, occasionally vomiting, especially in the morning; frequently the appetite was bad, the tongue furred, and there was constipation. The sleep was often disturbed by dreams. There was a general sinking of the corporeal and mental strength; dullness of the memory and of the power of th ught. The appearance was cachetic, and there were occasionally tremors and nervous weakness. It happened pretty constantly that the symptoms rapidly disappeared when the poisoned room was vacated for a while, or the arsenical substances were removed; they rapidly returned, however, when the patient reoccupied the room. Poisoning also occurred where arsenical paper had been covered over with paper that was free from this substance, or where the arsenic was present in oil colors. The author is of the opinion that arsenic is present in the air of such rooms in the form of arseniuretted hydrogen, and that it is more probably absorbed into the human body by way of the skin than by the respiratory organs.—*Nordiskt Med. Archiv.*

*Dysentery Cured Without Opium.*—Dr. J. H. Carstens (*Detroit Review of Medicine*) gives the following treatment and cases:

Frank H., æt. 15, was attacked with dysentery July 3d. This was during the height of the epidemic we have just passed. For him I prescribed one gr. of quinine, three drops of chloroform, and three drops fluid ext. ergot, every three hours. The bloody stools disappeared the next day, with but slight tenesmus the day thereafter, and in six days was entirely cured.

Henry O., aged three years, has had dysentery for two weeks; bowels moved every five minutes, the mother states. I had some suppositories made as follows: ℞. Pulv. ipecacuanhæ, ʒss.; pulv. ergotæ, gr. xv.; quiniæ sulph., gr. iv.; olei theobrom, q. s. For twelve small rectal suppositories.

I directed the mother to introduce one every two hours. The next day great improvement was noticed in the patient. I prescribed twelve more suppositories, which cured the child entirely by the second day, with no other treatment whatever but a good nutritious diet.

Charley K., aged two and one-half years, was brought to my office by his father, who gave the following history: The child had been suffering with a diarrhœa for some weeks, but in the last three days blood appeared in the stools, the child having, during the discharge, much tenesmus. I gave the father some of the above described suppositories, to be used as before stated. Calling at the house the next day, the mother stated that some of the suppositories were passed immediately after their introduction, and that they caused much bearing-down pain. I directed that if a suppository was passed shortly after its introduction, another one should be used immediately, and that it should be passed high up. This was done, and although some suppositories were passed, the others remained; the blood first disappeared, then the discharge diminished, and the child was well the third day.

*Aconite in Headache.*—Dr. Fothergill (*British Medical Journal*, January 10, 1874) recommends the use of aconite in congestive headaches, attended with great cotemporary coldness of the hands and feet. The remedy dilates the peripheral blood-vessels, and so relieves the congested cerebral vessels.

*Bromide of Camphor.*—Bourneville (*Progrès Médical*) has submitted the efficacy of bromide of camphor to a severe test, by choosing as a field for his experiments a hospital for incurables. If it succeeded in these obstinate cases, still greater was the probability that it would act beneficially where the conditions were more favorable, and the illnesses of more recent origin. A patient in the Hospital de la Pitié, twenty-four years of age, suffering from acute rheumatism, was attacked by chorea in the left arm. He was cured in five days. The dose was sixty centigrammes (nine grains) daily, given in six dragées.

In the same hospital, a woman, aged twenty-two, was attacked by violent hysterical chorea, with hysterical vomiting. The dose given was first forty, and then sixty centigrammes, daily. Her cure was rapid.

A young woman, a patient in the Necker Hospital, suffering from induration, with insufficiency of the mitral valve, showed symptoms of poisoning from the first day digitaline was administered to her. The digitaline was discontinued, and bromide of camphor substituted. The heart-beats diminished in frequency and became regular. The medicine was relinquished, and the improvement obtained continued the same a fortnight later.

A man in the same hospital, presenting the same conditions, received equal relief.—*Med. and Surg. Reporter.*

*Nitrite of Amyl and Belladonna in Dysmenorrhœa, as sugggested by Dr. Peters, of New York.*—The method is, to administer belladonna in ordinary doses for several days previous to the occurrence of the menstrual flow, and when pain comes, to adminster by inhalation from two to six drops of the nitrite of amyl p. r. n. In one case a single drop of amyl was all that was required.

Dr. Sell remarked that he had been in the habit of administering nitrite of amyl by the mouth, and had obtained just as good results as he had obtained when the remedy had been inhaled. He prescribed it in one-drop doses combined with drachm doses of peppermint water, and repeated every half hour. In one case of dysmenorrhœa, and one only, he had used the nitrite of amyl, and in that case the patient was completely relieved of pain.—*New York Med. Record.*

*Fissures in Ano Treated with Iodoform.*—Dr. Francesco Parona (*Giornale Italiano delle Mal. Ven. e della Pelle,* October) gives his experieuce of iodoform in fissures of the anus, and strongly recommends it. He believes it acts in a great measure as a local anæsthetic, " which allays the spasm of the sphincter during defecation, while it favors cicatrization by neutralizing the irritating effects of fœcal matters which may remain on the ulcerated surface." It has also a direct healing action. Dr. Parona uses the iodoform as an ointment (one part to three of lard), and applies it on a small cylinder of charpie, of a size requiring but little force for its introduction. The charpie has the advantage that its filaments adapt themselves readily to the slight irregularities of the anal mucous membrane. The dressing is changed twice a day, and replaced after each motion, and in the majority of cases the pain and spasm caused by the fissure cease in a few days, and the patient is well in a relatively short time. Of four cases, of which details are given, the longest time was twenty days, while one of the patients had been ill four months previous to the treatment.—*Medical Times and Gazette.*

*Physician as a Witness.*—The Hon. John T. Hoffman, in an address recently delivered to the graduating class of the Albany Medical College, gives the following advice, which commends itself to every one at all interested not only in making an impression as an expert witness, but in saving his reputation as an ordinarily educated medical man :

"When you go upon the stand as a witness, go well informed, but not too full of learning in the matters about which you are to testify. Confine yourself to facts, and do not be forced into theories any further than you can help. Use the simplest language you can adopt, and omit as far as practicable purely professional terms and phrases. Speak English and not Latin, for the jurors are not ancient Romans. Remember that the witness-box is the place in which to tell the plain truth, not to display learning. Meet quick questions with slow answers. Answer long questions with few words. Above all, keep cool. * * *" These rules are very simple, are founded on the plainest common-sense, and yet how often are they violated !

*The Treatment of Fistulous Sinuses by the Elastic Ligature.*—Mr. Allingham, of London, in a recent article on the above subject, after ascribing to Prof. Dittel, of Vienna, the honor of having brought into general notice the elastic ligature, though the first applications seem to have been made by Dr. Silvestri, of Vicenza, states that he is convinced from experience that there are decided advantages in the elastic ligature over the knife in many surgical cases, and particularly in the treatment of fistulæ. He mentions its use in sixty operations, of which forty were for fistulæ in ano, five were for hemorrhoids, two for removal of scirrhous breasts, two for varicocele, and the rest for various other diseases. In none did any serious consequence follow the operation, and it was noticed that cases which had been operated upon by the ligature did much better than those which had been treated by the knife, though the patients were under the same hygienic influences. He was careful not to be partial in the selection of cases. He thinks that the probable advantages of the ligature over the knife in ordinary sinuses are:

1. The operation is commonly painless, and the subsequent suffering, if any, is usually very slight.
2. It is bloodless.
3. There is greater rapidity of cure.
4. The patient need not keep his bed, nor even his room, but may go into the air and drive or walk in moderation.
5. Its peculiar applicability to delicate patients, and those who have a phthisical tendency.
6. There is usually no anæsthetic required.
7. There is a minimum amount of suppuration.
8. And one may add that the ligature is often very advantageous as a supplement to the knife.—*Medical Press and Circular.*

*Typhoid Fever.*—Dr. Montgomery, of Chicago, recommends the following formulæ in typhoid fever: ℞. Potassæ chloratis, ʒ ii.; acid muriatic dil., ʒ ss.; syrup simplex, ℥ ss.; aqua, ℥ iv. M. S. Tablespoonful every three hours in water. When they require stimulating, the following: ℞. Strychnia sulph., gr. i.; acid nitric, ʒ i.; syrup simplex, water, aa ℥ ii. M. S. A teaspoonful every six hours.—*St. Louis Med. and Surg. Jour.*

*The Extraction of Foreign Bodies from the Ear.*—Dr. John Cleland, of Edinburgh, relates the following in a letter to the *Lancet:*

When I was demonstrator in Glasgow, a student came to me in much alarm, with a pea in the auditory meatus, which had been flung in a class-room, and had been driven far in toward the membrana tympani by the efforts of one of his fellow-students to remove it. It fitted sufficiently accurately that there was no space for the introduction of an instrument behind it. I had no instrument beside me but a straight needle for microscopic purposes. I touched the upper margin of the pea with this, endeavoring to introduce the needle behind the pea, when the pea rolled somewhat and receded. It then occurred to me to put the needle beneath the pea, and, to my great surprise, I had scarcely begun to insinuate the point of the needle when the pea flew out with great violence. Any one may repeat the experiment, as I have done, with a pea, a tube, and a needle, and may easily arrive at the explanation of what happened in the case which I have mentioned. If the needle be placed above the pea, it presses on the uppermost part of the sphere. By a movement of combined pressure and withdrawal the pea may then be made to roll outward for a little distance; but in this movement needle and pea move together, so that the point of the needle, continuing to press on the portion originally uppermost but now rolled outward, ceases immediately to act in a purely downward direction, and jerks the pea back toward the membrana as soon as the increasing pressure inward exceeds the resistance of friction against the floor of the tube. But if the point of the needle be placed below the pea, it raises it up; and if it has been insinuated in the smallest degree beyond the lowest part of the sphere, and be on a higher level than the handle, the pea tends to roll out over it. It is not, however, essential that the point of the needle be on a higher level than the handle, provided only that the needle be properly insinuated in; for as soon as the handle is depressed so far that the point is at all inclined toward the roof of the tube, the pea is placed between two inclined planes, from between which it is expelled with force.—*Med. and Surg. Reporter.*

*Diabetes.*—Tannin is now recommended in diabetes. Minimum doses, ten grains, maximum from twenty to thirty grains, repeated two to four times per diem.—*The Archives*, October, 1874.

*Treatment of Delirium Tremens.*—Dr. Kitchen gives the following treatment, in the *American Journal of Insanity:*

In the treatment of delirium tremens many points are to be taken into consideration, as the condition of the patient, the length of time the delirium has lasted, and the surroundings of the patient.

Our custom is to place this class of cases in a large room, well ventilated, with about one thousand cubic feet of space for each patient.

Usually, the patient is much fatigued on admission, and is in feeble physical health, and not infrequently there are complications, as bronchitis or pneumonia, and occasionally Bright's disease.

When no complication exists we give a tepid bath. The patient is put to bed, and usually a camisole is required to restrain him.

The usual, and perhaps better, treatment is at once to place the patient on liberal and nutritious diet, as beef-juice, cream, or essence, soups, milk, milk punch, egg-nog, etc.

If he is feeble, the reasons for giving stimulants are plain, though the delirium is caused by the same stimulant. Some recommend pure alcohol to be given instead of brandy, whisky, or even wine.

Of course, in administering stimulants to this class of patients, great and watchful care should always be exercised. The pulse is a safe guide, as stimulants should lower it and give it fullness. To quiet the tremors and restlessness, opium serves a good purpose, administered by hypodermic injection.

The treatment which in all probability is the most effective, is a generous diet, full doses of fluid extract of conium during the day, to control the muscular action, and during the evening hydrate of chloral, with tincture of hyoscyamus, the latter to be repeated until sleep is secured.—*Med. and Surg. Reporter.*

*Turpentine an Antidote for Phosphorus.*—It appears to be well established that turpentine combines with phosphorus both in the stomach and in the blood, forming an almost inert turpentine-phosphorus acid. One part of phosphorus is more than neutralized by one hundred of turpentine. The antidote should be given for several days. Fats, oils and milk must be avoided, as they dissolve the phosporus and increase its activity. A patient recovering from phosphorus-poisoning under the use of turpentine was killed by a dose of castor-oil given as a purge.—*Physician's Monitor.*

The Paris correspondent of the *British Medical Journal* writes that, at a recent meeting of the Academy, M. LeFort read his report on a paper by Dr. Lelièvre on a substitute for the ordinary linseed-meal poultice, which M. LeFort was charged to investigate. Dr. Lelièvre proposes the "fucus crispus," or Carraghean lichen, as it possesses the following advantages: It may be cut into thin plates of the size required, and, when steeped in hot water, it softens and swells in a few minutes. This new poultice has been tried by MM. Demarquay, Gosselin, and Verneuil in their respective hospitals, and they have pronounced it to be far superior to the linseed poultice; it keeps moist for more than sixteen or eighteen hours; it does not slip; is inodorous; does not readily ferment, nor does it soil the linen or bed of the patient. This new poultice is destined to render great service to the hospitals, ambulances, and particularly on board ship, where it is difficult to keep the linseed in a state of preservation.—*Boston Med. and Surg. Journal.*

*Treatment of Catarrhal Jaundice by Electricity.*—Dr. Gerhardt, of Berlin, first determines the position of the gall-bladder by percussion at the free border of the liver. This can often be detected by a small rounded prominence at the inferior convexity of the liver, and can easily be made to project. The electrode of a strong inductive electric machine is appiled at this point, while the other electrode is applied at the other side of the median abdominal line. Almost always, when the current is powerful, a gurgling sound can be heard, and very often the fæces resume their natural color, and the cure is effected.—*Ber. Klin. Wochenchr.*

*Horse-Hair for Sutures.*—Dr. Fayrer, in his "Clinical Observations in India," remarks that a well-selected white hair out of a hoise's tail is, in many respects, better than any suture hitherto devised; that from the tail of a white or gray horse is the best. He cannot tell why it should be so, but he finds the white better than the black hair. The matter, he says, may appear a trifle, but nevertheless is an important trifle; for if the alleged inconvenience and even danger from suppuration from the hemp and silk ligature, or the disadvantages of the wire, can be avoided, the subject is sufficiently interesting to be worthy of consideration.—*Practitoner.*

*Bulk of Salts in Solution.*—I have often received prescriptions containing, among other ingredients, one or two ounces of Epsom salts (or any other salt), to be dissolved in—say 6 to 8 fl. oz. of some liquid. Partly from the dose and from other circumstances, it was evident to me that most of the prescribing physicians had no very clear idea—rather none at all—of the increase in bulk which the salt would occasion. As it cannot be indifferent to the physician whether a certain active drug is taken in one-twelfth doses or one-eighth doses, and so on, I think it not superfluous to state the real increase in bulk of some salts in solution:

| | *In Weight.* | | *By Measure.* |
|---|---|---|---|
| Epsom salts, | 8 | parts are equal to | 5 parts. |
| Rochelle salts, | 8 | " " | 3 " |
| Bi-carb. of potassa, | 8 | " " | 3 " |
| Glauber salts, | 8 | " " | 3 " |
| Sugar, | 3 | " " | 2 " |
| " | 8 | " are contained in | 10 (correctly $9\frac{2}{3}$), *Syrup, U. S. Ph.* |
| Gum arabic, | 4 | parts are contained in | 10 *Mucilage, U. S. Ph.* |

This is sufficiently near for all practical purposes; as to the other salts, they are generally prescribed in such small quantities, that the increase in bulk practically amounts to nothing.—*Druggists' Circular.*

*A Strange Story.*—The Lancaster (O.) *Gazette* says: "Mrs. John Watchel, an old resident of this city, some twelve years ago ran a needle in her breast, and failed to have it extracted, as it never occasioned her either pain or inconvenience. A few days since, however, the lady felt a strange pricking sensation in a bunion on one of her feet, and upon examination found the point of a needle protruding from the excrescence. With but little trouble it was taken out, and it appears to be the same needle she lost in her breast a dozen years ago."

*Soft Chancres.*—As a local application in these, the *Memorabilien* recommends a solution of sulphate of copper in glycerin, thirty grains to the ounce. They are said to cicatrize rapidly under this treatment.

*New Liniment for Scabies.*—Dr. Clemens gives the following formula in the *Allg. Med. Central Zeit.*, December 9th, 1874:

℞. Arsenious acid, gr. j.; carbonate of potash, grs. xv.; spirit of soap, ʒiij.; spring water, ℥iij. M. The liniment to be rubbed twice daily on the parts affected.

It is said to be efficacious, and unattended with any trouble or inconvenience. The carbonate of potash is added to the arsenious acid to promote its solubility, and to prevent the too rapid drying up of the liniment. Dr. Clemens has used this application for the last five years, with the best results. Of cousse, disinfection of the clothes is useful; but where such cannot conveniently be carried out, the liniment lying on the body serves as an attraction to the acari, which, by reaching it from the clothes, perish in it. Very little of the quantity mentioned in the prescription is necessary, and the frictions have always proved harmless as far as the digestive organs are concerned. It does not hurt the youngest child.—*Medical and Surgical Reporter.*

*The Hydrate of Chloral to Arrest Putrefaction and Fermentation.*—The experiments of Dujardin, Beametz, and Hirne show that the hydrate has a certain power in arresting fermentation, for the formation of tactic acid in milk was prevented by it and when only a one-per-cent. solution was used. The decomposition of urine was likewise prevented by a weak solution, so that at the end of a month it had undergone no change. As a local application, the writers found that it was excellent for foul wounds, gangrene of the skin or mucous membranes, cancerous sores, phagedenic chancres, and the like. They found in all cases that it produced no pain, destroyed the fetid odor, cleansed the surface, and promoted cicatrization. It seemed, therefore, specially adapted for a local application in carcinoma uteri. Judging from its power of preventing the decomposition of urine, they recommend it as an injection (one per cent.) in those diseases of the bladder that are accompanied by rapid decomposition of the urine.—*L' Union Medicale.*

*Pityriasis Versicolor.*—For this disfiguring disease, Dr. Ravoth has used with success a weak solution of caustic potash; and in one instance, citric acid.

*Antagonism between Chloral Hydrate and Calabar Bean.*—Dr. John Hughes Bennett (*Brit. Med. Jour.*) presents the experiments from which the following conclusions are drawn. Hydrate of chloral modifies to a great extent the action of a fatal dose of extract of calabar bean, mitigating symptoms and prolonging life. In some cases, chloral clearly saves life from a fatal dose of calabar bean. This antagonism is limited by two conditions:

1. By the doses administered. More than a minimum fatal dose of extract of calabar bean destroys life, notwithstanding the administration of chloral.

2. By the interval of time between the administration of the two substances. There is great probability of saving life in those instances in which both substances are given almost simultaneously. This probability is diminished if the chloral be given five or eight minutes after the extract of calabar bean. But even in those cases in which death occurs after the introduction of both substances, the effects of the calabar bean are much less marked.—*Detroit Review of Med. and Pharm.*

*Nasal Catarrh.*—For that very annoying, obstinate and disagreeable complaint, post-nasal catarrh, Dr. Dobell believes in a combination treatment of, first, a medicated injection: ℞. Boracis, ʒj.; glycerin carbolat, ʒij.; sodæ bicarb, ʒj.; aquæ tepidæ, Oss. Sig. To be used night and morning. Second: A medicated snuff, to wit: ℞. Powdered camphor, tannic acid, white sugar, dry Welsh snuff, aa ʒj. Sig. Two or three times a day. Third: A lozenge, as—℞. Powdered camphor, gr. ij.; tannic acid, gr. ss.; hydrochlorate of morphia, gr. $\frac{1}{36}$; white sugar, gr. x.; powdered gum arabic, gr. ij. Lastly, rub behind the ears and back of the neck, two or three times a day, with camphor liniment.—*Medical and Surgical Reporter.*

*Treatment of Epistaxis.*—Dr. W. I. Wilson, U. S. A., reports the successful employment, in two cases of severe epistaxis, of a solution of the liquor ferri persulphatis, one part to three of water, introduced into the nostril in the form of spray, by means of the ether-spray apparatus. The bleeding was checked in a few seconds. *Philadelphia Medical Times.*

*Deafness Cured by the Extraction of Teeth.*—Mrs. A., aged about thirty-eight years, presented herself at my office about five months ago for the purpose of having all the superior teeth in her mouth extracted, for the purpose of obtaining an artificial plate. The teeth, seven in number, were extracted in the following order: right second molar, right second bicuspid, four incisors, and left canine. Immediately after the removal of the canine tooth, the patient remarked that she could hear now from her left ear. She had been *entirely* destitute of hearing in that ear for upwards of three years, having lost the faculty while attending church one Sabbath morning. While sitting in church she said she had felt a severe pain in the abdomen, thence passing up her left side to her face and ear, which lasted about ten minutes, and from that time she had been entirely deaf in that ear. She said that after the removal of the canine tooth a sensation followed like a current of air passing in at her ear, which lasted several moments; immediately afterwards she recovered her hearing. Upon examination of the teeth, I found them much decayed, and all effected with "alveolar abscess," especially the right molar and incisor teeth—the bicupsid and eye-tooth not so much. I have frequently seen the patient since, and her hearing continurs good.—*Luther Campbell.*

*Treatment of Persistent Neuralgia.*—Amongst the many remedies that have been tried for rebellious neuralgias, M. Denos, of the Hôspital de la Pitié, recommends the following combination as being frequently successful; and even in cases where it has failed, if tried again after the lapse of a short time, it may succeed. He first applies over the painful spot three or four mustard poultices, and then rubs into the reddened surface a liniment composed of— Oil of hyoscyanum, ℥iss.; laudanum, ʒss.; chloroform, ʒiss. M. *Journal de Medicine.*

*Improved Glycerin Cement.*—A cement of great value for many purposes, and capable of being used where resistance to both the action of water and to that of heat is required, is composed by mixing glycerin with dry litharge, so as to constitute a tough paste. For uniting the joints of steam pipes, and other similar applications, this preparation is said to be very satisfactory.—*Pharmacist.*

Dr. B. Powell, Houston, contributes to the Texas Medical Association reports of fourteen cases of cerebro-spinal meningitis treated with bromide of potassium and ergot—four by himself, one fatal, due, he thinks, to want of early recognition of the disease; three by Dr. L. Hudspeth, Houston, all recovered; and seven by Dr. J. J. Burroughs, Houston, one fatal after the second visit, which result, the doctor seems to think, could have been averted had he been sent for at the very commencement of the attack. This treatment was commenced by Dr. P. in the belief that the disease is essentially an inflammation of the meninges of the brain and spinal cord, and that "bromide of potassium constringes the vessels of the cerebral mass," while "ergot constringes the vessels of the spinal cord." We think the recommendations, now generally adopted, are based on fine principles, while the results in the cases referred to, in connection with cases elsewhere reported, justify their still more general adoption.—*Virginia Medical Monthly.*

*Epilepsy Cured by Excision of a Neuroma.*—At a recent meeting of the Clinical Society of London, Mr. Barwell related an interesting case of a soldier, aged sixty, who had lost an arm in India, in 1846, two years after which epilepsy set in. On examining the stump, a neuroma was found, any pressure on which produced a fit. These fits became at length so numerous, that Mr. Barwell was induced to cut down upon and remove the neuroma. This operation was performed August 7th, and at last accounts (October 24th) the man had had no more epileptic fits, and was much improved in appearance and intellect.—*Boston Med. and Surg. Jour.*

*Lime Glycerin for Burns.*—This remedy is as follows: Oxidi calc., 3 parts; glycerin, 150 parts; spts. æther chlor., 3 parts. This preparation has been used by Laub for several years with great success. Charpie is to be dipped in the mixture and placed over the burned surface; it is then covered with a thin sheet of gutta-percha, and then a layer of charpie is added, the whole to be surrounded with a loose bandage. It is very important that the charpie should be closely applied to the burned surface. The pain ceases almost instantly, and the sore heals very rapidly.—*Nordisk Med. Archiv.*

*Mr. Erichsen on American Surgery.*—On November 9th, Mr. Erichsen delivered an address in London on his impressions of American surgery during his late tour in this country. It is in general favorable, as may be judged from the following extract:

"Surgery in the United States certainly stands at a very high level of excellence. The hospital surgeons throughout the country have struck me as being alike practical, progressive and learned in a very high degree. In practical skill, and aptitude for mechanical appliances of all kinds, they are certainly excelled by no class of practitioners in any country. They are thoroughly up to modern surgery in its most progressive forms, and I have never met with any class of men who are so well read and so perfectly acquainted with all that is done in their profession outside their own country. It would be a great injustice to American surgeons for it to be supposed that surgical skill is confined to the large cities, or to the few. On the contrary, I know no country in which, so far as it is possible to judge from the contemporary medical literature, there is so widely diffused a high standard of operative skill, as in the country districts and more remote provinces of the United States. The bent of the mind of the American surgeon is, like ours, practical rather than scientific; in fact, there are the same mental characteristics displayed in him that we find here; the same self-reliance, the same practical aptitude, the same *curative* instinct, which leads him to consider his patient rather as a human being to be rescued from the effects of disease or injury, than as a scientific object to be studied for the advance of professional knowledge How, indeed, can it be otherwise than that there should be such a resemblance?—*Phil. Med. and Surg. Rep.*

*Bromine in Pertussis.*—Dr. Vogelsang, of Brel, in Switzerland, finds that one or two scruples of bromine, as much bromide of potassium, to a tumbler of hot water, placed in the room of a child suffering from whooping-cough, affords it great relief. The mixture should be renewed three or four times a day.

*Warts.*—Dr. Guttceit recommends rubbing warts, night and morning, with a moistened piece of muriate of ammonia. They soften and dwindle away, leaving no such white mark as follows their dispersion with lunar caustic.

*Cæsarean Section Successful for both Mother and Child.*—Dr. Jacobs, of Cologne, reports that a woman, æt. 42, multipara, was delivered two years ago with difficulty by the forceps. The conjugate diameter was barely two inches; the horizontal rami of the os pubis were so driven inwards that the symphysis pubis projected like a beak. A careful examination showed that she was suffering from osteomalacia of the pelvis. As the child was living, it was, after consultation, decided to perform Cæsarean section. The patient was placed under chloroform, and the abdomen was rapidly divided through the linea alba, care being taken to empty both bladder and rectum previously. The incision into the uterus struck upon the placenta, which was detached as far as was requisite to remove the fœtus. The placenta was removed immediately after the delivery of the fœtus, without any difficulty. The uterus was then compressed and squeezed downwards. No sutures were placed in the uterus, only in the abdominal walls, which were brought together in the usual manner. There was some sickness from the chloroform, which was arrested by the internal and external administration of ice and ice-water. Only a portion of the abdominal wound healed by first intention; the rest granulated. Dr. Jacobs

This is the third successful case that has been reported lately; two were by pupils of M. Depaul, who strongly advocates the immediate removal of the placenta and the non-use of sutures to the uterus. He also recommends the insertion of a long seton through the two wounds, and out through the vagina. This method was adopted in each of the cases of his pupils.—*Berliner Klin. Woch.*

*Copaiba in Dropsy.*—Dr. Falconer (*Canada Lancet*) feels confident that very few practitioners have given this drug a fair trial in ascites. Passing by cases of cirrhosis and chronic peritonitis, with their accompanying effusions, in which, after re-accumulation after tapping, he has succeeded in not only diminishing, but in completely removing the fluid by the use of copaiba, he refers to a case of ovarian dropsy, which he treated successfully by the drug. Copaiba is the diuretic, *par excellence*, and never fails in removing the serum through the kidneys, except in chronic albuminuria. It obviates the use of eleterium or gamboge, tapping, or ovariotomy, in many cases.—*Medical Examiner.*

*Differential Diagnosis between the Red Blood Corpuscle of Human and Certain of the Lower Animals.*—Dr. Richardson, of Philadelphia, says that it is possible to distinguish the blood of man from that of the pig, dog, ox, etc. Dr. Woodward replies, closing his article in the following language:

"In conclusion, then, if the microscopist, summoned as a scientific expert to examine a suspected blood-stain, should succeed in soaking out the corpuscles in such a way as to enable him to recognize them to be circular discs, and to measure them, and should he there find their diameter comes within the limits possible for human blood, his duty, in the present state of our knowledge, is clear. He must, of course, in his evidence, present the fact as actually observed, but it is not justifiable for him to stop here. He has no right to conclude his testimony without making it clearly understood, by both judge and jury, that blood from the dog, and several other animals, would give stains possessing the same properties, and that neither by the microscope, nor by any other means yet known to science, can the expert determine that a given stain is composed of human blood, and could not have been derived from any other source. This course is imperatively demanded by common honesty, without which scientific experts may become more dangerous to society than the very criminals they are called upon to convict."—*Medical Record.*

*Toothache Drops.*—The *Dental Cosmos* for November, 1874, publishes the following formulas:

1. ℞. *Chloroform,* Sydenham's laudanum, aa ʒii; tinct. benzoin, ℥i. 2. ℞. *Creasote,* chloroform, aa ʒii; Sydenham's laudanum, ʒiv; tinct. benzoin, ℥i. 3. ℞. *Oil of peppermint,* rhegalene, chloroform, aa ʒiii; camphor, ʒii. 4. ℞. *Chloral,* camphor, aa ʒi; morphia, gr. ii; oil of peppermint, ʒii.

*A Cure for Bright's Disease.*—Dr. Hegswald says a half pint thrice daily of a fresh infusion of the leaves of *Asplenium Scolopendrium,* L., is a most successful treatment in Bright's disease. This is the harts-tongue or spleenwort, and is said to be popular in Devonshire and elsewhere for its medicinal virtues.—*Phil. Med. and Surg. Rep.*

*Acidulated Gargles in Typhoid Fever.*—M. A. Netter, alluding to the buccal element in typhoid fever, and the beneficial influence of frequently-repeated acidulated gargles, draws the following conclusions:

1. Call the attention of the patient to the bad odor of their mouth, and inform them that not only in it, but also in the nose, there is something being secreted which poisons the whole system.

2. Place at their disposition an unlimited quantity of a solution containing two hundred grammes of decoction of barley, thirty grammes of honey, and twenty-five grammes of vinegar. Let them gargle and rinse their mouth with this frequently, and also snuff it into both nostrils. When they have commenced with this, it will be found so agreeable that large quantities will be consumed.

3. The nurses should be instructed to encourage and assist the patients during this operation. Where the adynemia is very profound, the nurses should cleanse the mouth for the patients.—*Gaz. des Hôp.* and *Trib. Méd.*, No. 308, 1874, G. R. C.

*Preparation of Sponge Tents.*—Paul Boyce, M. D., of Borden, Cal., in *Virginia Medical Monthly*, says: Having for the past two years met with *decided success* in the use of sponge-tents, it may be of interest to know how I prepare them. The formula I adopt is, first, to make a saturated infusion of hydrastis canadensis, to which is to be added enough of gum acacia to make the infusion of the proper consistence; then, for every one dozen tents to be prepared, I add—℞. Acid carbolici, gr. xxiv; ol. caryophylli, gtt. xj. *Misce bene,* and add to the infusion. Then proceed as usual in the manufacture of tents—insert the wire, wind, trim when dry, and coat with gelatin.

The propriety of using the above will be apparent at once. Tents thus made are the best I have ever used.

*Toothache Drops.*—Dr. Q. C. Smith praises the following most highly (*London Med. Record*): Take of carbolic acid, saturated solution, chloral hydrate, saturated solution, paregoric, fluid extract of aconite, of each an ounce; of oil of peppermint half an ounce; saturate the pledget of cotton or a piece of sponge, and tightly pack in the cavity.—*Phila. Med. Times.*

*Ailanthus Glandulosa in Dysentery.*—Dr. Cobert, medical chief of the British navy in China, extols the bark of the root of this tree as superior to ipecacuanha, or any other drug, in the treatment of dysentery. It is intensely bitter, like quinia, and produces vomiting when freely used. Dr. Robert found the dried bark of the root as good as the recent. The Chinese physicians who employ it give a cup of the strong infusion twice a day. The tree grows luxuriantly in all parts of the United States, having been introduced for the purpose of shade. It is a very rapid grower, and propagates itself abundantly by shoots from the root, being almost a nuisance in this respect. The tree is quite common in California, and is known as the Ailanthus, or Pride of China. Some further account of its remedial application may be found in the *American Journal of Pharmacy* for June, 1874.—*Pacific Med. Journal.*

*A Remedy for Toothache.*—The folllowing recipe I have prescribed for toothache, and have never found it fail. Should you deem it worthy reproduction in the *Reporter*, please insert it: ℞. Chloral hydrat., ʒj.; aqua pura, f℥ss.; misce, et adde tinct. aconit. rad *Flem.* gtts. xv.; chloioform pura, ether. sulph., aa. gtts. xx.; sp. vini rect., f℥ ss. M. Ft. mistura. Sigma. Rub the gums with a little of the mixture; put a few drops upon cotton, and insert in any cavity of the tooth. Also take the following dose on sugar: For an adult, fifteen to thirty drops; for a child, two to ten drops, according to age. The above doses can be repeated quite frequently, if necessary.

*Guaiacum in Acute Diseases of the Throat.*—Dr. Fritzinger, of Ashland, Pa., in the *Med. and Surg. Rep.*, extols guaiacum in the treatment of acute tonsolitis and other inflammatory affections of the throat. He thinks it acts first as a local astringent; second, a diaphoretic; third, a laxative; and fourth, a specific resolvent on the inflamed tonsil glands. He prefers the following formula: ℞. Potassæ chloratis, ʒj.; spt. eth. nit., ʒiv.; tinct. guaiaci, ℥iss. M. Sig. A teaspoonful every three hours in sweetened water. It is an old practice revived, with the exception that the powder was formerly used instead of the tincture.—*Pacific Med. and Surg. Reporter.*

*Hypodermic Injection of Ergot in Post-partum Hæmorrhage.*—Dr. P. C. Williams, of Baltimore, in a paper read before the Medico-Chirurgical Faculty of Maryland, 1874, recommends the hypodermic injection of the fluid extract of ergot in post-partum hemorrhage. He gives in detail three cases, in which he has found its use most advantageous in stopping severe flooding. In two of the cases it was not tried until other remedies had been employed to no purpose. The effect of the hypodermic was almost instantaneous, and it was permanent. The fluid was injected in the inside of the thigh, and in no case was an abscess produced.—*Obstetrical Journal.*

*Preparation of Koumiss.*—Three quarts of fresh milk, one-fourth pound grape sugar, and fresh beer-yeast of the size of a hazel-nut, are mixed, heated upon a slow fire to 25° R. (88° F.), removed from the fire for a short time, then again heated as before, at once filled into champagne bottles to within an inch of the neck, and these well corked. The bottles should be agitated every fifteen minutes during the next forty-eight hours. If well prepared, Koumiss must effervesce like soda water.—*Allg. Med. Centralzeitung,* 1874, p. 1108.

*Elegant Ferruginous Preparation* (Prof. Goodell).—The following offers simply the most elegant and efficient ferruginous preparation we know of:

Take of tincture of the chloride of iron three fluid drachms, dilute phosphoric acid half a fluidounce, syrup of lemons three fluidounces. M. A whitish preparation, pleasant to the taste; to be exhibited in a dose of a dessertspoonful to a tablespoonful.—*Philadelphia Medical Times.*

*Cardiac Œdema.*—In a lecture in La Charite, on disease of the heart, Professor See advocated the following treatment of Œdema and anasarca due to heart affections: Extract of squills, fifteen grains; powder of squills, ten grains; for ten pills. From six to ten of these pills to be taken daily. At the same time about one drachm of bromide of potassium is administered daily. Under the influence of these two drugs the dropsy is stated to diminish rapidly.—*Med. and Surg. Rep.*

*Glycerin-Siohel.*—The *Gazette Obstet. et Gaz. de Joulin,* according to *La Tribune Medicale,* states that this preparation, which consists of pure glycerin and the yelk of egg, and has the appearance and consistence of honey, is highly praised as a local application for several purposes. In fissures of the nipple of nursing women it affords relief when other remedies fail. It afforded relief in every one of eleven such cases in which it was applied. In fissures of the mammæ, it protects the skin from the action of the salvia of the child, and from sour milk. In ruptures of the perineum, it protects the torn surfaces from being irritated by the urine and lochia. It is daily used in Dr. Vernier's clinics to annoint the hands when examining suspicious women, and is found to be a perfect protection from infection. This preparation does not putrefy, nor does it become rancid like ointments. It consists in cleansing wounds, and promotes primary union.—*Am. Jour. Med. Sci.*

*Catheter Impacted in the Female Pelvis.*—At a recent meeting of the Royal Medical and Chirurgical Society, Mr. Barwell reported the case of a female whom he found suffering from a large abscess over the hip, with a sinus in front of the anus, all of which seemed to have originated in an instrumental abortion produced upon her twenty months previous. A subsequent examination, made after the abscess had been evacuated, revealed the presence of a gum-elastic catheter, lying up between the uterus and bowel. A small opening was detected communicating with the rectum, through which the catheter was forced, and then drawn out at the anus.

*A Fly of Africa.*—There is a Cayenne fly, called the *Lucilia Hominivorax* (man-eater), which commits great havoc among the convicts sent out to that colony by the French government. M. Charles Coquerel says that this fly lays its eggs in the mouth or nostrils of a sleeping convict, especially a drunken one, and that the offspring in their larval state usually bring about the death of their victim.—*Popular Science Monthly.*

FROM recent experiments, Professor Lister has arrived at the conclusion that the septic matter present in water is not in solution, but consists of insoluble particles, which are held in suspense.

